# cGAS-STING signaling pathway in lung cancer: Regulation on antitumor immunity and application in immunotherapy

**DOI:** 10.1016/j.pccm.2024.11.001

**Published:** 2024-12-12

**Authors:** Jing Wu, Yingyao Chen, Mengqing Xie, Xin Yu, Chunxia Su

**Affiliations:** Department of Oncology, Shanghai Pulmonary Hospital & Thoracic Cancer Institute, Tongji University School of Medicine, Shanghai 200433, China

**Keywords:** Cyclic GMP-AMP synthase-stimulator of interferon genes (cGAS-STING), Lung cancer, Tumor microenvironment

## Abstract

The innate immune system has a primary role in defending against external threats, encompassing viruses, bacteria, and fungi, thereby playing a pivotal role in establishing robust protection. Recent investigations have shed light on its importance in the progression of tumors, with a particular emphasis on lung cancer. Among the various signaling pathways implicated in this intricate process, the cGAS-STING pathway emerges as a significant participant. Cyclic GMP-AMP synthase (cGAS) discerns free DNA and activates the stimulator of interferon genes (STING), subsequently culminating in the secretion of cytokines and exerting inhibitory effects on tumor development. Consequently, researchers are increasingly interested in creating anticancer drugs that specifically target the cGAS-STING pathway, offering promising avenues for novel therapeutic interventions. The objective of this review is to present a comprehensive overview of the ongoing research on the cGAS-STING signaling pathway within the realm of lung cancer. The primary emphasis is on understanding its involvement in lung cancer development and assessing its viability as a target for innovative therapeutic options.

## Introduction

Immunotherapy has transformed the therapeutic landscape of advanced lung cancer, substantially prolonging overall survival and enhancing the quality of life for afflicted patients. Despite these advancements, a considerable number of patients exhibit primary or acquired resistance to immunotherapy, posing a significant clinical challenge. This resistance is often attributed to the complex interplay between tumor cells and the immunosuppressive microenvironment, which undermines the efficacy of immune-based treatments.^1^ Consequently, there is an urgent need to identify novel therapeutic strategies capable of circumventing resistance mechanisms and improving patient outcomes.

The immune system operates through two main branches: innate immunity and adaptive immunity. While current immunotherapeutic strategies predominantly target adaptive immunity, recent research underscores the critical role of innate immunity in shaping the antitumor immune response.^2^ One pivotal pathway garnering increasing attention is the cyclic GMP-AMP synthase (cGAS)-stimulator of interferon genes (STING) pathway, which serves as a vital regulator of innate immunity with profound implications for cancer immunotherapy.^3^ Within the intricate immune microenvironment of lung cancer, characterized by a delicate balance of pro-inflammatory and immunosuppressive signals, the cGAS-STING pathway emerges as a central orchestrator governing the dynamic interplay between innate and adaptive immunity. Primarily, cGAS activates innate immunity and exerts an antitumor effect by detecting cytoplasmic DNA and triggering the production of interferons (IFNs) and pro-inflammatory cytokines. Additionally, cGAS-STING facilitates the maturation of dendritic cells (DCs), enhances antigen presentation, and augments the antitumor efficacy of adaptive immunity.^4^

Understanding the regulation and dysregulation of the cGAS-STING pathway in lung cancer provides invaluable insights into tumor progression and immune evasion mechanisms. Also, it holds immense potential for advancing innovative therapeutic strategies, precise diagnostics, and improved patient outcomes.

## Overview of cGAS-STING signaling pathway

The discovery of the cGAS in 2013 marked a significant milestone in our understanding of DNA recognition and innate immunity.^5^ Here, we provide a comprehensive summary of the cGAS signaling pathway, elucidating its intricate molecular mechanisms and downstream effects. cGAS possesses two DNA binding domains: facilitating efficient recognition and binding to cytoplasmic DNA. Under normal physiological conditions, the cytoplasm maintains minimal levels of DNA, resulting in the inactivation of cytoplasmic DNA receptors associated with cGAS.^6^ However, during specific stress conditions, the cytoplasmic DNA content increases, leading to the activation of cGAS.^7^ Furthermore, cGAS harbors a highly conserved nucleotidyltransferase (NTase) domain responsible for catalyzing the synthesis of cyclic GMP-AMP (cGAMP) from guanosine triphosphate (GTP) and adenosine triphosphate (ATP).^8^ Upon interaction with DNA, cGAS undergoes a conformational change and engages in enzymatic activity, generating cGAMP as an endogenous second messenger. Beyond its role in intrinsic cellular signaling, cGAMP produced by cGAS can also elicit a broader regional immune response. This response involves the activation of neighboring cells and immune cells in the vicinity, leading to the establishment of an antiviral and immunomodulatory environment.

Once synthesized, cGAMP acts as a signaling molecule by binding to the STING protein, which is an endoplasmic reticulum (ER)-associated transmembrane protein encoded by the transmembrane protein 173 (*TMEM173*) gene.^9^ The binding of cGAMP induces a conformational transition within the STING dimer, leading to its activation.^10^ Activated STING then undergoes translocation from the ER to the Golgi apparatus, a crucial step in propagating the signal downstream.^11^

Within the perinuclear region, STING orchestrates the recruitment and subsequent phosphorylation of TANK-binding kinase 1 (TBK1). Upon activation, TBK1 phosphorylates the carboxyl-terminal structural domain of STING, facilitating the recruitment of interferon regulatory factor 3 (IRF3). Phosphorylated IRF3 forms dimers and translocates to the nucleus, where it initiates the transcriptional activation of *IFN* genes.^12^ The newly expressed type I IFNs then activate Janus kinase 1 (JAK1), leading to the assembly of the signal transducer and activator of transcription 1/2 (STAT1/2) heterodimer and interferon-stimulated gene factor 3 (ISGF3) complex.^13^ This complex translocates into the nucleus, activating the transcription of interferon-stimulated genes (ISGs), which encompass a broad array of antiviral and immune-related genes.

**Fig. 1:**
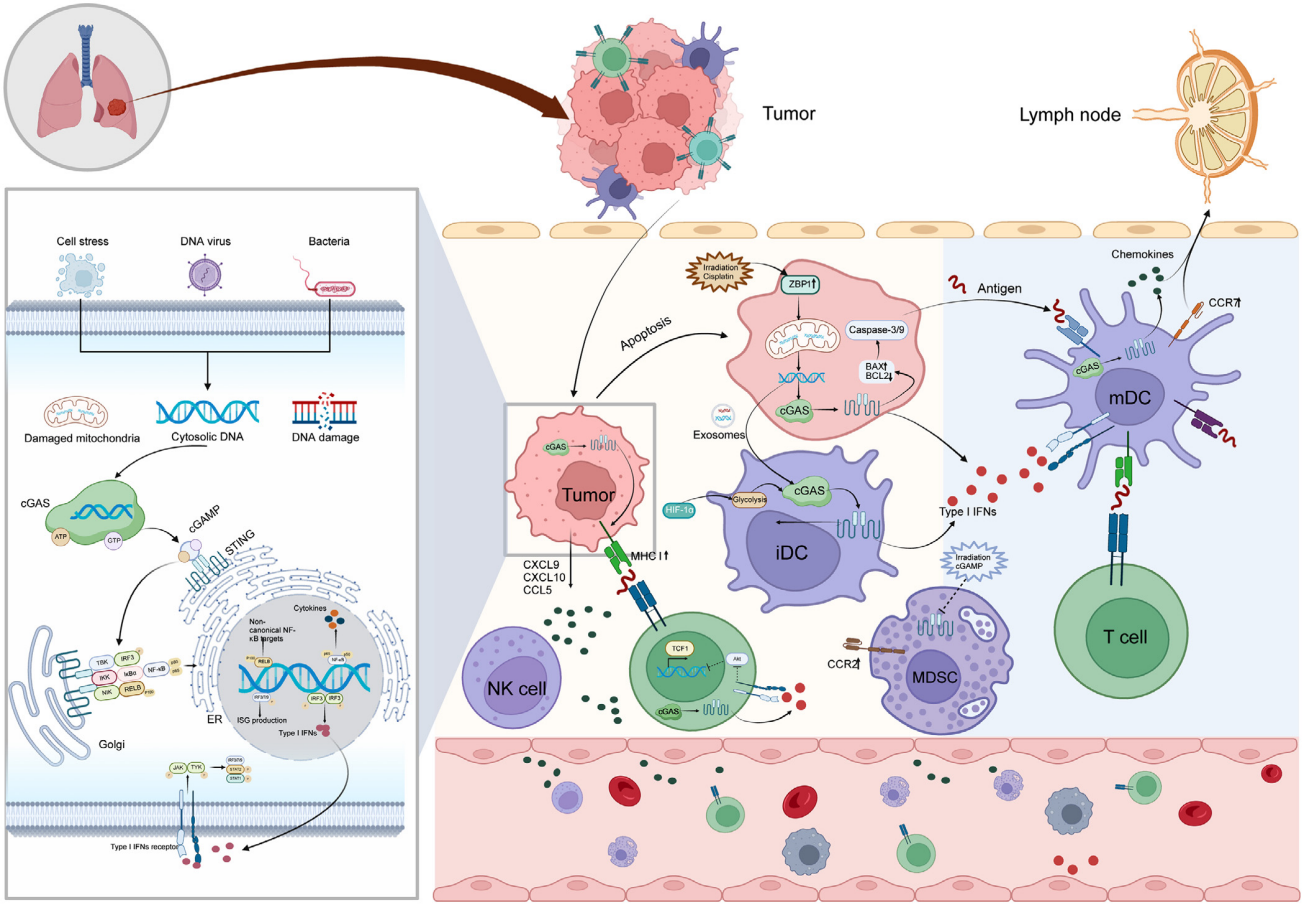
cGAS-STING tumor suppression mechanism. Upon activation of cGAS by cytosolic DNA, the synthesis of 2′3ʹ-cGAMP and subsequent binding to STING triggers a signaling cascade that results in the activation of transcription factors such as IRF3 and NF-κB. This activation induces the expression of genes involved in the immune response, including type I IFNs and cytokines. The intricate cascade ultimately leads to the activation of immune cells, prompting them to recognize, attack, and eliminate tumor cells, offering a promising direction for antitumor therapy. AKT: Protein kinase B; ATP: Adenosine triphosphate; BAX: BCL2 associated X; BCL2: B-cell lymphoma-2; cGAMP: Cyclic GMP-AMP; cGAS: Cyclic GMP-AMP synthase; CCL5: C-C motif chemokine ligand 5; CCR2: C-C motif chemokine receptor 2; CCR7: C-C motif chemokine receptor 7; CXCL9: C-X-C motif chemokine ligand 9; CXCL10: C-X-C motif chemokine ligand 10; GTP: Guanosine triphosphate; HIF-1α: Hypoxia inducible factor 1 subunit alpha; iDC: Immature dendritic cells; IFNs: Interferons; IκBα: Inhibitor kappa B-alpha; IKK: IκB kinase; IRF3: Interferon regulatory factor 3; ISG: Interferon-stimulated gene; mDC: Mature dendritic cells; MDSC: Myeloid-derived suppressor cells; MHC I: Major histocompatibility complex I; NF-κB: Nuclear factor kappa-B; NIK: Nuclear factor-κB-inducing kinase; JAK: Janus kinase; NK: Natural killer; RELB: RELB proto-oncogene, NF-kB subunit; STAT: Signal transducer and activator of transcription; STING: Stimulator of interferon genes; TBK: TANK-binding kinase 1; TCF1: T cell factor-1; TYK: Tyrosine kinase; ZBP1: Z-DNA binding protein 1.

In addition to the TBK1-mediated signaling cascade, STING also plays a role in activating the nuclear factor kappa-B (NF-κB) pathway. The exact mechanism by which STING promotes NF-κB signaling is still under investigation, but studies have proposed the involvement of TBK1 and TNF receptor associated factor 6 (TRAF6)-NF-κB signaling axis.^14^ STING can activate mitogen-activated protein kinase kinase kinase 14 (MAP3K14)^15^ and inhibitor of kappa B kinase (IKK),^16^ triggering the classical and non-classical NF-κB signaling pathways, respectively. These pathways lead to the transcriptional upregulation of pro-inflammatory cytokines and immune-related genes, further amplifying the immune response.^17^ After termination of signal transduction, it is transferred to lysosomes for degradation (Fig. 1). Recent research has strongly demonstrated the intricate involvement of the cGAS-STING signaling pathway in diverse cellular processes, including tumor cell senescence, autophagy, apoptosis, and the preservation of immune-related cell stemness.

## Role of the cGAS-STING pathway in regulating antitumor immunity in lung cancer

The cGAS-STING pathway plays a pivotal role in regulating tumor cell senescence and apoptosis and bolstering antitumor immunity, making it a critical component in antitumor mechanisms.^18^ In this section, we will focus on the research progress concerning the pathway's involvement in regulating the growth of lung cancer.

### Regulating tumor cell senescence and apoptosis

Cellular senescence is characterized by a state of prolonged growth arrest, leading to cell cycle exit and cessation of proliferation. Senescent cells exhibit a distinctive secretion profile, releasing various cytokines, including inflammatory cytokines, growth factors, and proteases, collectively termed as the senescence-associated secretory phenotype (SASP).^19^ The release of DNA by tumor cells can induce senescence by activating the cGAS-STING signaling pathway, triggering senescence through the NF-κB signaling cascade or SASP factor secretion.^20^ After exposure to irradiation or oncogenic transposons, cGAS- and STING-deficient mice show decreased upregulation of SASP factors like interleukin 6 (IL-6), C-X-C motif chemokine ligand (CXCL) 10, and cyclin dependent kinase inhibitor 2A (Cdkn2a) *in vivo.*^21^ Furthermore, nuclear programmed death-ligand 1 (PD-L1) silencing in the lung cancer A549 cell line enhances STING promoter activity, upregulates STING expression, induces tumor cell senescence, and inhibits lung cancer growth. The study has shown that combining PD-L1 silencing with senescence-inducing chemotherapy drugs has demonstrated significant efficacy in inhibiting tumor growth.^22^ These findings underscore the pivotal role of the cGAS-STING pathway in regulating cellular senescence. Activation of this pathway may contribute to the amplification of inflammation in senescent cells, as SASP typically occurs as a late event in the senescence process. However, it is important to note that in this chronic inflammatory state, cytokines secreted by senescent cells, such as interleukin 1 beta (IL-1β), IL-6, and matrix metalloproteinase (MMP), continue to be released, potentially promoting tumorigenesis.^23^ Chronic inflammation instigated by senescent cells, which release cytokines such as IL-1β, IL-6, and MMP, can exacerbate tumorigenesis. Studies have demonstrated that mutations in *Kras/p53* enhance the release of mitochondrial DNA (mtDNA), thereby activating cGAS-mediated inflammation and fueling the progression of lung cancer. Baicalein has been shown to curb mtDNA release and interact with cGAS, thereby inhibiting its activation and mitigating cGAS-mediated inflammation in the context of lung cancer development.^24^ These studies provide new perspectives for understanding immunogenic death-initiating antitumor immune responses and new targets for enhancing tumor therapy.

### Enhancing antitumor immunity

cGAS recognizes cytoplasmic DNA, promoting the formation of cGAMP, which in turn activates STING. This activation leads to the induction of a diverse array of pro-inflammatory cytokines and chemokines, along with the maturation of DCs. Additionally, it enhances the capacity for tumor-associated antigen (TAA) presentation and stimulates a specific CD8^+^T cell response against tumors. This multifaceted mechanism holds significant promise for bolstering tumor immunity and overcoming drug resistance. In this subsection, we present an overview of the research progress in this area specifically focusing on lung cancer.

#### DCs

DNA fragments released by cancer cells in the tumor microenvironment (TME) are internalized by DCs through exosomes.^25^ Upon entry, these DNA fragments activate the cGAS-STING pathway, leading to the production of type I IFNs that drive the maturation and activation of DCs. Additionally, cGAS-STING pathway activation within DCs retards lysosomal acidification, impeding the clearance of tumor antigens. This activation also elevates the expression of major histocompatibility complex class I (MHC-I) molecules on the DC surface, facilitating the presentation of antigens to T and B lymphocytes, a critical step in mounting an effective immune response against the tumor.^26^ Studies have revealed that enhanced hypoxia-inducible factor 1-alpha (HIF-1α)-mediated glycolytic metabolism in DCs treated with the STING agonist cGAMP amplifies their antitumor efficacy, with elevated glycolysis further promoting STING signaling in human non-small cell lung cancer (NSCLC), creating a positive feedback loop.^27^ Upon activation of the cGAS-STING signaling pathway, mature DCs migrate to the draining lymph nodes in response to chemokines, where TAAs are presented to T cells. *In vitro* studies have shown that C-C motif chemokine receptor 7 (CCR7) expression on DC cells co-cultured with type I IFN promotes DC homing migration and migration to tumor-draining lymph nodes.^28^ In conclusion, the findings from these studies provide compelling evidence demonstrating that the STING pathway enhances the ability to regulate antigen presentation in DCs, thereby exerting its antitumor immunity.

#### CD8^+^T lymphocytes

STING activation promotes the secretion of chemokines, such as CXCL9, CXCL10, and C-C motif chemokine ligand 5 (CCL5), establishing a chemical gradient that guides cytotoxic T lymphocytes (CTLs) into tumor tissue. Before T lymphocytes can effectively recognize and eliminate tumor cells, they must first extravasate and migrate from the circulatory system to the TME. The cGAS-STING pathway plays a pivotal role in facilitating this process, orchestrating T lymphocyte infiltration and trafficking into malignancies through various mechanisms. Notably, recent research has underscored the clinical significance of cGAS-STING activation in NSCLC, revealing a correlation between enhanced expression of cGAS, CCL5, and CXCL10 and a favorable prognosis in patients with NSCLC undergoing chemotherapy and immunotherapy.^29^ Additionally, due to their own mitochondrial dysfunction, *KRAS-LKB1* (KL) mutant NSCLC cells exhibit cytoplasmic accumulation of mtDNA. This leads to epigenetic silencing of STING expression through enhancer of zeste homolog 2 (EZH2)-mediated methylation of histone H3 at lysine 27 (H3K27) and DNA methyltransferase 1 (DNMT1)-induced accumulation of 5-methylcytosine around its promoter, thereby protecting cells from STAT1-induced cytotoxicity. Deletion of STING signaling in tumor cells in KL cells impairs the infiltration of cytotoxic T cells into the TME, creating resistance to anti-PD-1 therapy.^30^ Furthermore, small cell lung cancer (SCLC) inflammation is a subtype characterized by the absence of typical transcription factors but specifically expresses numerous immune checkpoint (IC) and human leukocyte antigen (HLA) genes. Studies have demonstrated that T cell-attracting chemokines such as CCL5 and CXCL10 induced by the STING are expressed at high levels in this subtype.^31^ However, lung cancer also evades immune surveillance by inhibiting the cGAS-STING signaling pathway through various mechanisms. Metabolic reprogramming, particularly glycolysis, is a significant biological marker of lung cancer. NOP2/Sun RNA methyltransferase 2 (NSUN2), a novel glucose-interacting protein, stabilizes three prime repair exonuclease 2 (TREX2) after mediating glucose binding, limiting cytosolic double-stranded DNA (dsDNA) accumulation, cGAS/STING activation, and CD8^+^ T cell infiltration. This promotes tumorigenesis and leads to resistance to anti-PD-L1 immunotherapy.^32^

In addition to stimulating DCs, activation of the STING pathway enhances the recognition and clearance of cancer cells by CD8^+^ T lymphocytes. The upregulation of MHC-I expression in cancer cells triggers CTL-mediated cancer cell death.^33^ Interestingly, Mahadevan *et al*^34^ identified a unique subset of SCLC characterized by the upregulation of MHC-I, which exhibits durable benefits from immune checkpoint blockade (ICB). Furthermore, they demonstrated epigenetic restoration of MHC-I in SCLC following the loss of neuroendocrine differentiation in an *in vitro* model, suggesting a role for the disinhibition of STING in this process. Additionally, the STING pathway plays different roles in various stages of lung cancer. When lung adenocarcinoma cells transition from a relatively quiescent (nonproliferative) dormant phase to the proliferative phase, DNA replication increases, leading to STING activation. Subsequently, STING activation triggers surrounding immune cells to mount attacking the cancer cells.^35^ Meanwhile, recent studies have demonstrated a positive correlation between cGAS-STING activation and improved prognosis in chemotherapy and immunotherapy for various cancer types, including NSCLC, colorectal, cervical, breast, and melanoma cancers.^36^ However, high doses of STING activators can cause the activation of non-type I IFN domains, disrupting calcium homeostasis and rendering T cells hypersensitive to ER stress induced by T cell receptor (TCR) signaling.

T cell therapy stands out for its remarkable specificity and potent antitumor effects, countering tumor evasion mechanisms. Yet, prolonged antigen exposure often leads to T cell exhaustion and reduced function, urging the development of long-lasting reprogrammed T cells. The decreased expression of cGAS and STING in peripheral blood CD8^+^ T cells of cancer patients underscores the crucial role of these molecules in CD8^+^ T cell function. Studies utilizing a mouse tumor model of T cell reinfusion confirm that deficiencies in cGAS or STING within CD8^+^ T cells diminish their antitumor efficacy. Activation of the endogenous cGAS-STING pathway in CD8^+^ T cells inhibits protein kinase B (AKT), thus preserving T cell factor-1 (TCF1) expression, fostering stem cell differentiation, and bolstering the persistence and expansion of transferred T cells.^37^ This preservation of stem-like CD8^+^ T lymphocytes ensures a robust and enduring T cell population capable of effectively combating tumors. Such insights hold substantial implications for T cell therapy, emphasizing the pivotal objective of enhancing the effectiveness and persistence of CD8^+^ T lymphocytes in immunotherapy approaches.

#### Natural killer (NK) cells and myeloid-derived suppressor cells (MDSC)

NK cells are indispensable components of innate immunity, pivotal in surveilling tumors and virus-infected cells. Activation of the cGAS-STING pathway in non-tumor cells by NK cells triggers a potent NK cell response, culminating in tumor elimination—a phenomenon known as “bystander effect”. This mechanism significantly bolsters the overall antitumor immune response within the TME.^38^ Moreover, overexpression of exonuclease 1 (TREX1) hampers STING-dependent nucleic acid sensing in KL lung cancer cells, impeding NK cell recruitment and reducing sensitivity to NK cell-derived IFNγ by degrading cytoplasmic DNA.^39^ Notably, MET-amplified lung cancer exhibits resistance to immunotherapy, with studies revealing weakened STING signaling in these patients. This results in reduced proportions of CD8^+^ T cells and NK cells, accompanied by increased expression of exhaustion molecules in both cell types, ultimately compromising immune cell function.^40^ Exogenous cyclic dinucleotides, which activate the cGAS-STING pathway, have shown promise in bolstering NK cell activation and eliciting antitumor effects in mouse models of colorectal cancer and melanoma.^41^

MDSCs constitute a heterogeneous population of immature myeloid cells known for their pivotal role in dampening T-cell immune responses, thereby facilitating immune evasion in tumor environments. The majority of studies have indicated that both intrinsic and extrinsic STING signals in MDSCs are associated with a decrease in MDSC abundance, contributing to a more favorable immunological milieu within the TME.^42^ However, intriguingly, investigations focusing on lung cancer have presented conflicting findings. In a notable study, CCR2^+^ monocytic MDSCs (M-MDSCs) were observed to accumulate in MC38 colon cancer and lewis lung carcinoma (LLC) tumors in a STING-dependent manner following tumor irradiation with a single dose of 20 Gy or intratumoral injection of the STING agonist cGAMP.^43^ This finding suggests that STING signaling may play a role in the recruitment of M-MDSCs to the tumor site, potentially exacerbating immunosuppression.

## Modulation of the TME by the cGAS-STING pathway

Antigen-specific immune recognition and clearance of cancer cells rely on the infiltration of effector T cells into the TME, facilitated by vascular endothelial cells. However, tumor vessels often exhibit malformations and dysfunction, characterized by a disorganized network of immature microvessels with reduced cellular support. Consequently, hypoxia and acidosis in the TME induce the production of immunosuppressive factors that hinder T-cell homing, extravasation, and antitumor effector functions.^44^ STING signaling plays a pivotal role in normalizing tumor vasculature, enhancing pericyte coverage, and preserving a more intact basement membrane. This is achieved through the secretion of type I IFNs and the upregulation of vascular stabilization genes such as *Angpt1, Pdgfrb*, and *Col4a*. Consequently, STING signaling promotes the intratumoral infiltration of effector CD8^+^ T cells and mitigates hypoxia within the TME.^45^ Meanwhile, Lv *et al*^46^ found that the tumor suppressor gene *TET2* in hepatocellular carcinoma upregulates cGAS expression in cancer cells, activating STING in endothelial cells. This activation leads to tumor vascular normalization and enhances lymphocyte recruitment and trans-endothelial migration, thus providing a rationale for combining STING-based immunotherapy with antiangiogenic therapy. Furthermore, activation of the STING pathway in endothelial cells upregulates the synthesis of adhesion molecules, including intercellular adhesion molecule 1 (ICAM-1), vascular cell adhesion molecule 1 (VCAM-1), and E-selectin. These molecules facilitate the extravasation of T lymphocytes from blood vessels and promote their infiltration into tumors.^47^ This recruitment enhances the efficacy of immune cells in targeting metastatic lung cancer cells and inhibiting metastatic spread.

In mouse models of breast cancer, lung cancer, and melanoma, administration of low doses of STING agonists like cGAMP and ADU-S100 has demonstrated a synergistic effect in promoting tumor-associated vascular normalization, recruiting circulating immune cells, and facilitating endothelial adhesion.^48^, ^49^, ^50^ This process enables the infiltration of tumor-infiltrating lymphocytes into the TME, laying the groundwork for the formation of tertiary lymphoid structures (TLS). TLS formation occurs at specific sites through the interaction of lymphoid tissue inducer cells (LTi) and lymphoid tissue organizer cells (LTo).^51^ Studies suggest that type I IFN can induce the expression of CXCL13, attracting B cells and initiating the development of ectopic germinal centers. CXCL13 further transforms non-lymphoid tissues into functional TLS structures under the influence of other stimuli.^52^^,^^53^ These therapeutic effects correlate with the coordinated formation of non-classical TLS and the emergence of a distinct tumor-infiltrating TCR repertoire within the TLS-positive TME, which is undetectable in the peripheral immune cell compartment. Overall, these findings suggest potential avenues for modulating the TME and shaping the antitumor immune response, although further research is needed to determine the optimal approach.

## Utilizing the cGAS-STING pathway for antitumor immunotherapy

With the elucidation of the mechanism of STING action and the progress of translational medicine research, agonists targeting STING have emerged as a promising agent for the development of antitumor drugs. These agonists encompass various types, including vadimezan, cyclic dinucleotides and their derivatives, and small molecule agonists, as detailed in Table 1.^54^ STING agonists have shown promising preclinical benefits in lung cancer. These agonists can enhance antigen presentation, stimulate the synthesis of pro-inflammatory cytokines, and increase NK cell activity. These effects ultimately result in tumor cell death and the subsequent initiation of adaptive immune responses.^55^ However, using STING agonists as monotherapy in lung cancer presents challenges. Direct intertumoral injection of STING agonists has been observed to have undesirable effects on the TME. This treatment approach may result in a rapid upregulation of IC molecules, including PD-L1, as well as enzymes like cytochrome c oxidase subunit II (COX2) and indoleamine 2,3-dioxygenase 1 (IDO).^56^ These molecules are associated with immunosuppression. Consequently, this modulation of the immune response can give rise to a “cold” TME, exhibiting insufficient infiltration of immune cells and diminished antitumor immune activity.^57^ Therefore, combining STING agonists with other appropriate antitumor therapies can produce a synergistic effect in mechanisms.

**Table 1:** The clinical development of STING agonists in lung cancer.

Compound type	STING agonist	Route of delivery	Indication	Combination	Clinical trial phase	Results/state	Clinical trial NCT Code
DMXAA	DMXAA	Intravenous injection	aNSCLC, second line	Docetaxel	Phase III	Terminated	NCT00738387
			aNSCLC, first line	Paclitaxel and carboplatin	Phase III	ORR: 25% mPFS: 5.5 months mOS: 13.4 months	NCT00662597
			SCLC, first line	Paclitaxel and carboplatin	Phase II	ORR: 94% mPFS: 7.0 months mOS: 14.2 months	NCT01057342
			aNSCLC, first line	Paclitaxel and carboplatin	Phase II	ORR: 46.7% mPFS: 5.5 months mOS: 14.9 months	NCT00832494
CDNs and derivatives	ADU-S100	Intratumoral injection	Advanced solid tumors or lymphomas	Spartalizumab	Phase I	Terminated	NCT03172936
				Ipilimumab	Phase I	Terminated	NCT02675439
	MK-1454	Intratumoral injection	Advanced solid tumors or lymphomas	Pembrolizumab	Phase I	ORR: 0%	NCT03010176
	SB 11285	Intravenous injection	Advanced solid tumors	Atezolizumab	Phase I	Recruiting	NCT04096638
	IMSA101	Intratumoral injection	Refractory malignancies	Geptanolimab	Phase I/II	ORR: 66%	NCT04020185
	BMS-986301	Intratumoral/intramuscular injection	Advanced solid tumors	Nivolumab/ipilimumab	Phase I	Active, not recruiting	NCT03956680
	E7766	Intratumoral injection	Advanced solid tumors or lymphomas	–	Phase I	Terminated	NCT04144140
Non-CDNs	TAK-676	Intravenous injection	aNSCLC, TNBC, SCCHN	Pembrolizumab	Phase I	Active, not recruiting	NCT04879849

aNSCLC: Advanced non-small lung cancer; CDNs: Cyclic dinucleotides; mOS: Median overall survival; mPFS: Median progression free survival; ORR: Overall response rate; SCCHN: Squamous cell carcinoma of head and neck; SCLC: Small cell lung cancer; STING: Stimulator of interferon genes; TNBC: Triple negative breast cancer; SCCHN: Squamous cell carcinoma of the head and neck; -: Not available.

### STING agonist combined with chemotherapy/radiotherapy

Chemotherapy serves as the standard treatment for lung cancer, exerting its effects by interfering with the DNA synthesis and repair mechanisms within cancer cells. This disruption leads to the accumulation of DNA damage, which is detected by the cGAS sensor, subsequently activating the STING signaling pathway. This activation triggers downstream effectors to produce type I IFN, thereby initiating an immune response against the tumor. In combination with chemotherapy, STING agonists can synergistically enhance the release and presentation of tumor antigens, bolstering the antitumor immune response. DMXAA, identified in 2002, was the first-generation STING agonists.^58^ Although it displayed promise in initial clinical trials,^59^ subsequent phase 3 trials yielded unsatisfactory results, leading to its discontinuation due to poor outcomes.^60^ Further research revealed that DMXAA's effectiveness in humans was hampered by species differences in the STING receptor. To address this limitation, second-generation STING agonists like MSA-2 have been developed, offering improved stability and the potential for systemic administration. Preclinical studies combining these second-generation STING agonists with chemotherapy agents, such as 5-fluorouracil (5-FU), have demonstrated enhanced anticancer effects and reduced toxicity.^61^ Despite these promising findings, research on the combination of STING agonists with chemotherapy in lung cancer remains limited. Additional investigations are warranted to fully elucidate the therapeutic potential of this treatment modality for patients with lung cancer.

Radiotherapy induces lethal DNA damage directly in irradiated cells or indirectly through the generation of reactive oxygen species.^62^ This damage includes base mutations, single-strand breaks, and double-strand breaks, leading to chromosome instability and micronucleus formation.^63^ Additionally, radiotherapy triggers oxidative stress and mitochondrial dysfunction in cancer cells, resulting in alterations in mitochondrial membrane permeability. This allows mtDNA to exit the mitochondria and enter the cytoplasm through mitochondrial permeability transition pores or BCL2-associated X protein (BAX)/BCL2 antagonist/killer (BAK)-dependent mitochondrial outer membrane permeability.^64^ Recently, combination therapy with 2′5′-cGAMP and irradiation has shown promising results in enhancing antitumor responses, leading to tumor volume reduction, increased survival rates in mice, and elevated production of type I IFN in lymph nodes.^65^ However, whether radiotherapy can induce a cGAS-STING-mediated antitumor effect depends on the applied radiation dose. After 12–18 Gy irradiation, DNA exonuclease Trex1 is produced in different cancer cells, which degrades cytoplasmic DNA, leading to the reduction of dsDNA required for cGAS/STING activation and inhibiting the antitumor immune effect.^66^ Further research is needed to fully understand and optimize the therapeutic potential of combining radiotherapy with cGAS-STING pathway modulation for cancer treatment.

### STING agonist combined with DNA damage response (DDR) inhibitors

The application mechanism of DDR inhibitors in lung cancer involves blocking the DNA damage repair pathway of tumor cells, thereby increasing the accumulation of DNA damage, and inducing tumor cell death.^67^ Prior *in vivo* investigations have revealed that the application of poly ADP-ribose polymerase (PARP) inhibitors and checkpoint kinase 1 (CHK1) inhibitors can effectively obstruct the DDR pathway, which exhibits substantial activity in SCLC. Further mechanistic investigations have revealed that the treatment modalities induce the release of inflammatory factors by activating the STING pathway, thereby exacerbating tumor cell death. On the contrary, the suppression of cGAS and STING diminished the antitumor effects observed when combining DDR inhibition with anti-PD-L1 therapy.^68^ Hence, combining DDR inhibitors with STING agonists represents a potential strategy to enhance immune responses in SCLC treatment.

### STING agonist as a cancer vaccine adjuvant

STINGVAX, in combination with granulocyte-macrophage colony-stimulating factor (GM-CSF), is an innovative STING-based tumor vaccine.^69^ In preclinical studies, the administration of STINGVAX demonstrated an augmentation in lymphocyte infiltration within tumors, as well as upregulation in PD-L1 expression. Moreover, the combination of STINGVAX and a PD-L1 blocker showed superior effectiveness when compared to monotherapy with the PD-L1 blocker alone.^69^

In another research, therapeutic vaccination against metastatic breast cancer utilizing cyclic di-guanosine monophosphate (c-di-GMP), a STING ligand, facilitates the upregulation of TAA Mage-b and the activation of specific T cells to exert antitumor effects.^70^ Furthermore, the combined utilization of c-di-GMP and an attenuated vaccine expressing TAA demonstrated a notable reduction in the concentration of MDSCs within the bloodstream. Consequently, this combinatorial approach effectively lessened the TME immunosuppression and impeded the progression of the main tumor and metastatic lesions.^70^ The presence of MDSCs and immunosuppression is common in many malignancies, indicating the chance of c-di-GMP in other types of tumors, including lung cancer.

### STING agonist combined with immune checkpoint inhibitor (ICIs) therapy

The emergence of ICIs has revolutionized lung cancer therapy by leveraging the immune system's ability to target cancer cells. However, a significant portion of patients fail to achieve long-term benefits from initial treatment or experience relapse after an initial response.^71^ This highlights the need for novel approaches to enhance immunotherapy efficacy, such as combining ICIs with other therapies like STING agonists. Preclinical studies have shown that concurrent administration of STING agonists with ICIs inhibits tumor growth and overcomes resistance to PD-1 therapy.^65^ Several STING agonists combined with ICIs, such as MK-1454^72^ and BMS-986301,^73^ have shown promising outcomes in clinical trials, including notable reductions in lesion volume and significant tumor regression. Additionally, ongoing research aims to evaluate the potential of ICIs combined with STING agonist derivatives, including ADU-S100, BI1387446, and E7766, for cancer treatment.

### STING agonist combined with chimeric antigen receptor T (CAR-T)-cell therapy

In recent years, CAR-T therapy stands out as a highly promising cellular immunotherapy, with notable advancements made in the treatment of diverse malignant tumors. By utilizing the single-chain variable fragment domain, CAR-T cells possess the ability to specifically identify and bind to the designated antigen,^74^ leading to the destruction of the cancer cells.^75^ Despite its potential, the effectiveness of CAR-T cell therapy in lung cancer is constrained by immunosuppression, physical barriers, and antigenic heterogeneity existing within the TME.^76^ Additionally, the heterogeneity of lung cancer cells can lead to some cells not expressing the target antigen, making them resistant to CAR-T cell therapy.^77^ PARP inhibitors modulate the TME and, via the cGAS-STING pathway, modify the equilibrium of immune stimulation signals. This modulation enhances the infiltration of CAR-T cells and facilitates the induction of tumor regression even at low doses of CAR-T cells.^78^ Moreover, the utilization of STING activators, including DMXAA and cGAMP-like agonists, exhibited augmented tumor regulation by CAR-T cells in *in situ* breast cancer models.^79^ These findings suggest exciting new opportunities to enhance the potency of CAR-T cell therapies within the domain of lung cancer.

## Perspective and conclusion

In recent years, the advancement of treatment strategies targeting adaptive immune responses has notably improved the outlook for patients with lung cancer. However, despite the pivotal role of the innate immune system in cancer immunity, our comprehension of its intricacies remains limited. The cGAS-STING pathway has emerged as a critical link between innate and adaptive immunity, facilitating tumor suppression through the secretion of type I IFNs. Nonetheless, recent findings suggest a nuanced role for this pathway, as autonomous activation by cancer cells can confer resistance, highlighting the exploitation of conserved inflammatory signaling to counteract drug stress.^80^ Consequently, numerous complexities within this pathway necessitate further elucidation. Delving deeper into the cGAS-STING pathway holds promise for guiding drug development and offering novel clinical perspectives in lung cancer treatment.

The future outlook for the cGAS-STING pathway in the field of lung cancer is promising, as it plays a crucial role in tumor immune surveillance and could serve as a target to enhance the efficacy of immune checkpoint inhibitors, thereby improving immune responses in lung cancer patients. Additionally, research on the activity of the cGAS-STING pathway may lead to the identification of new biomarkers for predicting treatment response and disease prognosis. Exploring the combination of the cGAS-STING pathway with other immunotherapeutic approaches, such as CAR-T cell therapy and vaccines, may further enhance treatment efficacy. Deepening our understanding of the specific mechanisms of the cGAS-STING pathway in lung cancer cells can provide a theoretical foundation for new therapeutic strategies. Furthermore, personalized treatment plans based on the status of the cGAS-STING pathway could improve the precision and effectiveness of lung cancer therapy, along with the development of drugs targeting this pathway to activate it and boost the immune system's attack on lung cancer. Overall, the cGAS-STING pathway holds significant potential for providing new insights and strategies for lung cancer treatment. These strategies aim to optimize the use of STING agonists in cancer therapy.

## Declaration of competing interest

The authors declare that they have no competing interests.
